# The Epistemic Value of Affective Disruptability

**DOI:** 10.1007/s11245-021-09788-5

**Published:** 2021-12-14

**Authors:** Imke von Maur

**Affiliations:** Institute of Cognitive Science, Department of Philosophy of Cognition, Osnabrück University, Osnabrück, Germany

**Keywords:** Epistemic practice, Habits, Disruption, Social epistemology, Affective intentionality

## Abstract

In order to explore how emotions contribute positively or negatively to understanding the meaning of complex socio-culturally specific phenomena, I argue that we must take into account the habitual dimension of emotions – i.e., the emotion repertoire that a feeling person acquires in the course of their affective biography. This brings to light a certain form of alignment in relation to affective intentionality that is key to comprehending why humans understand situations in the way they do and why it so often is especially hard to understand things *differently*. A crucial epistemic problem is that subjects often do not even enter a process of understanding, i.e., they do not even start to consider a specific object, theory, circumstance, other being, etc. in different ways than the familiar one. The epistemic problem at issue thus lies in an unquestioned faith in things being right the way they are taken to be. By acknowledging the habitual dimension of affective intentionality, I analyze reasons for this inability and suggest that being affectively disruptable and cultivating a pluralistic emotion repertoire are crucial abilities to overcome this epistemic problem.

## Introduction

The Civil war gave me the experience that epistemologies are not something abstract to be given over only to historians of science; epistemology creates the kind of world that we live in and the kind of human values that we have. (Varela 1979: 15)

To decide what is epistemically valuable we need to decide what kind of knowledge community is desirable, and this can’t help but involve political priorities and political choices. (Haslanger 2012: 354)

The refugee “crisis” led to an intensely discussed and constantly changing collective affective dynamic – anger, hatred, and burning refugee accommodations, the great shock when faced with the picture of the dead body of the three-year-old Aylan Kurdi who fled from war and drowned in the sea, the enthusiastic “welcome culture”, and (alleged) fear which discharged in anger and hatred against “the foreign”. From 2016 onwards, Brexit and Donald Trump dominated the international newspaper headlines for several years – equally filled with emotional upheavals of opposing pro/contra camps, the latter one finding its peak in the 2021 storming of the United States Capitol with an angry crowd screaming for their supposed rights and a shocked crowd worried about democracy. Any emotion involved in these socio-cultural settings both presupposes and leads to specific assumptions about reality. The situations are disclosed affectively in their (intra- and interindividual) meaningfulness in very different ways. There actually seems to be “no sensemaking overlap” (Protevi 2009: 44) between the realities of pro- and contra Brexit camps, between Trump voters and Trump opponents or between those believing in and those denying the existence of the coronavirus. This indicates that one crucial problem regarding epistemic positions and practices of socially situated individuals lies in the inability to *understand the realities of others*.^1^ Departing from this observation, this paper aims at shedding light on one crucial aspect that explains this inability and on potential ways to overcome it. Rather than primarily focusing on the cognitive or so-called rational underpinnings of this inability and of ideological tunnel-vision more broadly, I take serious John Protevis call for considering what he calls “affective ideology” and his underlying worry that most often the realm of what is considered ideology is confined “too much to the strictly cognitive, so that it is limited to epistemic problems with beliefs, propositions, concepts and the like” (Protevi 2016: 358). The key tenet of my approach, on the contrary, is, that the realities I described above are disclosed *affectively* based on internalized *historically and socio-culturally specific values and norms* that belong to a larger net of practices making up the everyday lives and identities of humans. The meaningful Gestalts they disclose and the “little worlds” they inhabit not only shape the space of possibilities for what can and cannot be understood, but under specific circumstances, they also prevent subjects from commencing a process of understanding in the first place. The paper is structured as follows: In *section two,* I introduce the concept of *habitual affective intentionality* as the way in which humans disclose meaningfulness. The crucial point of this concept and the related notions of *meaningful Gestalts* and *little worlds* is that the content and context of emotions must be understood in a practice-specific manner to reveal a structural intertwining of emotions with a certain kind of alignment. This alignment is the topic of the *third section,* in which I sketch two ways emotions become epistemically problematic. First, they attach the subject to alignments that are necessary to uphold their practices and forms of living and thereby make it impossible for them to enter understanding processes. Second, the habituality can become solidified and “ankylozed” (Al-Saji 2014) so that no other than the already familiar meaningful Gestalts can be disclosed, with the consequence that other possible ways of understanding remain a blind spot for the subject. In the *fourth section*, I elaborate on how far affectivity, in turn, can become epistemically *valuable* as a means to counter these problems. Affectivity is necessary to allow for the openness prevented by attaching to alignments and by making invisible other possible meaningful Gestalts. Accordingly, an affectively experienced disturbance of alignments is the precondition for the possibility to engage in understanding processes, and an affective disruption enables transformation of habitual affective intentionality and solidified emotion repertoires.

## Habitual Affective Intentionality

The concept of *habitual affective intentionality* brings together three crucial areas for an investigation of emotions: emotions’ world-disclosive dimension (intentionality), their socio-cultural embeddedness in a concrete context (situatedness) and their belonging to a diachronically developed emotion repertoire (habituality). In this paper, I build upon this conceptual framework, which I developed elsewhere, to reveal how emotions contribute negatively as well as positively to the aim of (commencing) understanding (processes) involving complex epistemic objects. Such processes might for instance involve objects like “racism”, “the ecological crisis” or “the Holocaust”, they might be about another person, another way of living or identity, or other complex phenomena that a subject engages epistemically. In this section, I introduce habitual affective intentionality as the means by which meaningful Gestalts are disclosed and “little worlds” are inhabited.^2^ This paves the way for elaborating how this way of disclosure is connected with certain alignments and how this connection poses an epistemic problem.

Emotions are intentional phenomena, which means they present the world as being meaningful in a certain way. Importantly, though, emotions do not track single features of the objects they are directed towards, but rather disclose a complex and encompassing *meaningful Gestalt*. Thus, emotional content is not reducible to single evaluative properties like “amusing” or “disgusting” but includes the concerns of the individual against the background of their socio-culturally specific situatedness. A person’s fear of blundering their talk at a conference is only intelligible against their background concern of giving a good performance. Similarly to how Gestalts are disclosed visually in reversible figures (like the famous duck-rabbit made prominent by Wittgenstein), in an emotional disclosure, the individual at the same time *receives* certain information and *construes* its meaning (Roberts 2003). This is why “disclosure” should be taken as a performative rather than merely a receptive notion: The complex meaningful Gestalt is brought into existence – importantly not only for the feeling person but for their environment as well. When the academic is ashamed because they made a mistake in their talk, they do not privately experience the situation as shame-worthy but rather construe a reality – what I call a “little world” – that is shared with the audience. The situational context with its affective space of possibilities is different from one in which they might have reacted with amusement. The notion of a “little world” is drawn from María Lugones’ (1987) introduction of “worlds” to denote the navigation through multiple ways of being from a phenomenological perspective. She describes, for instance, the different “worlds” of herself and her mother or the difference between the “worlds” of “being a Latina” and “being a stereotyped Latina”. Importantly, inhabiting a world is experienced in a specific way and demands a certain (affective) engagement that differs from inhabiting other potential worlds. Lugones’ notion of “worlds” helps to highlight the normative structuring experienced by differently situated bodies and is adopted in my approach to denote the concrete context in which subjects disclose meaningful Gestalts. A little world, on my account, is a concrete reality (at a given time and place) that is normatively structured with respect to a specific practice, which in turn refers to a form of living.

Affectively construing meaningful Gestalts and inhabiting “little worlds” allows the feeling subject to navigate through everyday practices. Such “aligned affectivity” orientates and allows unreflective yet skillful coping (Rietveld 2008). The crucial thesis is that humans are *affectively aligned* to certain practice-specific norms. A specific emotion repertoire is acquired during one’s affective biography *through which* one discloses certain meaningful Gestalts but not others. A famous example by Maurice Merleau-Ponty (1976: 193–194) gets to the heart of the embodiment of practice-relative normativity I aim to highlight: A soccer player moves on the soccer field in an unreflective yet skillful manner responding adequately to the field-specific norms. Importantly, the player has embodied a “sense for the field” as a concrete space, but also for the “game”, i.e., the movements of the other players, and their meaning, and the actions they need from the player. Adapting this to the social realm: Subjects navigate through social spheres accordingly and respond skillfully to “field specific norms”. Over time they internalize and embody the norms, which become second nature and the habitual schemata *through which* the subjects make sense of their worlds. I construe affective habitualization during the affective biography similarly. In a “paradigm scenario” (de Sousa 1987) a child learns the meaning of an emotion – for instance that a specific feeling it has is called “anger”. During their ongoing affective biography, the individual enacts further meaningful Gestalts in relational processes with their socio-cultural environment. The subject reacts affectively in given situations and thus has specific emotional dispositions at hand, and thereby certain meaningful Gestalts rather than others. To illustrate the embodiment of practice and life form-specific norms and the way in which this enables and restricts the space of affectability and the emotion repertoire, consider the following example:

*Alex enjoys the first spring sun while shopping in Berlin. They are in the capital for an internship, but right now it’s the weekend: leisure time. Alex already came across a variety of hip shops, bought trendy clothes, and tested a fancy kale smoothie. While imagining, with a huge smile on their face, how to combine the new clothes and what to wear for the party tonight with their colleagues, Alex passes an impressive arrangement of grey blocks of stone. They feel the need to take a picture and share it on Instagram. A yoga pose, that would look great – Alex thinks. And in the next moment, they ask a person to take a picture of them on the stone, one leg behind and the arm to the front. “Awesome!” Alex thinks happily, puts a hashtag below the picture, and clicks “share”. Filled with feelings of urbanity, creativity, inspiration, and freedom, and a thrill of anticipation of the many likes and comments the picture will receive, Alex continues their shopping trip through Berlin.*

The emotion Alex experiences in front of the grey stones is only understandable against the background of their socio-cultural-specific emotion repertoire. They do experience the objects described relating to the practice of posting things on social media. Their grandmother would have certainly reacted with completely different emotions towards the same socio-materiality, for the very practice of posting did not even exist for her. Importantly, the different ways of disclosing meaningful Gestalts as well as thereby opening up “little worlds” for oneself and others occur not as such – they are the ways in which we are aligned, in which our everyday engagement works in a flow, undisturbed without recognizing us being affected and affecting *in these ways*. To understand the following epistemic point, it is important to note that the way meaning is disclosed via affective intentionality is at the same time the product and the producer of socio-cultural practices and thus forms of living. If emotion repertoires are for instance racist, sexist, transphobic, or in any other way discriminatory, it becomes especially vivid why a normative evaluation of affective intentionality has to take this circumstance into account: It matters *which* little worlds we disclose (together) and which practices and forms of living we thereby bring into existence. What becomes apparent here is that the epistemic is bound up with the ethical and political, and that it is difficult to separate these spheres from one another (see José Medina (2013) for an argumentation along these lines and Karen Barad (2007) for establishing the notion of “ethico-onto-epistem-ology”, who thereby also considers the materialization of ethico-epistemic practices).

## Epistemic Problems of Habitual Affective Intentionality

I begin investigating the epistemic implications of this framework by elaborating on how habitual affectivity poses a *problem* for the epistemic practices of feeling subjects that do not come into view if the habituality of affective intentionality in its socio-cultural specificity is ignored. In the subsequent section, I move on to discuss affectivity’s epistemic *value* to counter exactly these problems.^3^

### Unassimilable Objects and Passionate Attachments

Consider the following example from Ágnes Heller (1981: 62): A five-year-old child watches a movie about the Second World War and classifies fascists as “bad people”. This fits nicely in the child’s “own world”, where there is already a place for “bad people”. As a six-year-old, the child reads a book in which a fascist is depicted who is very kind and lovely to his own son. The child is seized with a negative feeling – their world does not fit anymore. This negative feeling, described by Heller, is an example of what I call “affectively experienced disturbance of alignment”. An affectively experienced disturbance of alignment marks a break in (affective) interaction that is usually experienced as harmonic. The normative structuring of reality obstructs the skillful habitual engagement – the world does not respond in the familiar way. The child is disturbed by an “unassimilable object”^4^ and this disturbance is experienced negatively – it manifests a “directed discomfort” (Rietveld, 2008) concerning the source of disturbance that does not fit in. The affectively experienced disturbance is negatively valenced and thus provides a motivation for sustaining or restoring the stability of the familiar reality. Accordingly, the child will seek to escape the negatively experienced disturbance (Heller 1981: 62). It can, for instance, call the book stupid and reject its depiction of reality. The book just portrays things wrongly. This strategy allows the child to hold on to their habitual, familiar, aligning categories about “good” and “bad” humans – their own world stays intact (ibid.). The epistemic problem at issue thus occurs *before* concrete evaluation of an object takes place. In order to contour this problem more sharply consider the following scenario:

*While flipping through TV channels, Anniken happens upon a report about the working conditions of seamstresses in a textile factory in Cambodia. The pictures are unbearable, the seamstresses' working conditions violate human dignity. An unpleasant feeling overtakes Anniken the moment she recognizes that her favorite clothing brand produces its wares in this very factory. She turns away from the TV and thinks: ‘Well, the people have a job after all. If I didn’t buy the clothes produced there, the seamstresses would not be able to work and earn money to survive.’ The negative feeling ceases and she changes the channel.*

If Anniken would be confronted with new and plausible arguments about some matter with which she had little or no affective involvement, for example how photosynthesis works, she would probably not hesitate to revise the false beliefs she held about it until then. But being *a passionate fashion blogger*, she is unable to revise her belief that the clothes she buys and advertises are produced in innocuous circumstantial – even in the face of new and plausible arguments that tell her otherwise. This suggests the thesis that especially *those* beliefs that carry meaning for a person are hard to revise. Such beliefs (as the belief in the innocuousness of textile industry or the belief that humans are either good or bad) can – against one’s better judgment – not be abandoned because they are *cherished beliefs*, as Jason Stanley writes:

A cherished belief […] is a belief that “we want to retain; it is a pleasant belief to hold” [… A]n individual’s *emotional attachment* to a belief is what makes it difficult to rationally revise […]. (2015, 196)

Insights about photosynthesis and a change in her epistemic position regarding this epistemic object are not affectively experienced by Anniken. However, she is highly affectively involved when it comes to the production conditions of the clothes she buys and advertises. Importantly, it is not affectivity *as such* that poses the epistemic problem here. The problem is not that the affective involvement “skews” what could have been seen clearly in a supposedly non-affective manner. Rather, the epistemic problem the present paper aims at bringing to light results from an adherence to “affective alignments” which enable and sustain specific practices and forms of living. Such “passionate attachments” (Butler 1997: 6ff.)^5^ make it impossible for the subject to engage in understanding processes concerning complex epistemic objects. Accordingly, my thesis is that *one* crucial epistemic problem of habitual affectivity is that specifically aligned patterns of thinking, feeling, and acting cannot be called into question straightforwardly, because the subject is affectively attached to them due to their need for a stable reality. Thus, the affectively experienced appreciation of beliefs qua attachment to personal concerns – like Anniken’s belief in the innocuousness of the textile industry – is not an intrinsic property of such beliefs, as Stanley (2015: 197) correctly notes. Rather, we can only understand and explain the fact that these beliefs are worthy of being cherished against the backdrop of *cherished practices* and self-ascribed identities. Thus, it is misleading to concentrate on inner psychological flaws of emotional information processing, for instance, to approach the question of why humans cling to beliefs despite having good reasons to call them into question or revise them and thus do not even enter an understanding process regarding these beliefs. That humans are existentially attached to their aligned affectivity results from the fact that the loss of alignment might mean the loss of cherished practices and forms of living – or in extreme cases even the loss of *any* alignment leading to a Kafkaesque loss of orientation (psychosis). This shows that there are indeed reasons for the fear of the new or foreign and the loss of alignment that should be taken seriously. The relevant and severe epistemic problem lies in the *pre-reflective resistance to any calling into question of one’s own practices and forms of living* and thus in the one-sidedness of disclosed Gestalts, and, further in the inability to engage with new information which does not fit in the familiar alignment(s) – i.e., not allowing such information to enter a process of understanding. The habitual pre-reflective affective intentionality of the fashion blogger differs from that of someone not engaged in the practice of fashion blogging but who campaigns for Amnesty International. Both developed different ways of being affected by the very same epistemic object “textile industry” because of their specific affective biographies. They disclose – because of their specific emotion repertoires – completely different meaningful Gestalts.

The attempt to explain the epistemic problem of the reading child or the fashion blogger with reference to a missing “knowing, that” or with reference to a misrepresentation of concrete evaluative properties of a concrete object fails. Rather, the epistemic failure which results from an attachment to affective alignment lies in the inability of humans to engage in open and holistic understanding processes. In the cases discussed so far, it is not possible to determine binarily if something is understood or not. The epistemic phenomena coming into view here concern a temporary blocking in an inevitable and continuously ongoing stream of intertwined feeling, thinking, interpreting, acting, etc. – a hermeneutical circle out of which there is no escape, but within which a subject can either move or get stuck. The phenomenon of attachments to affective alignment is multifaceted and encompasses beliefs, desires, emotions, etc., which are held together by a narrative *through which* the feeling person discloses their reality. This will be made explicit in the next section.

### From Habitual Affective Intentionality to “Affective Ankylosis”

The disclosure of meaningful Gestalts via habitual affective intentionality means that a person makes sense of the world according to incorporated schemata. In this way, a person is aligned to what is familiar and what is available within their emotion repertoire. Still, subjects are able to switch between *different possible Gestalts*, as Robert Roberts importantly notes:

A person at whom I am inclined to be angry may be regarded, quite at will, in various ways: as the scoundrel who did such-and-such to me, as the son of my dear friend so-and-so, as a person who, after all, has had a pretty rough time of it in life, and so forth. If these construals are all in my repertoire, and in addition are not too implausible with respect to the present object, then the emotions that correspond to them, of anger, affection, and pity, are also more or less subject to my will. (2003: 81)

Although this demonstrates that habitual affective intentionality involves potential openness, there *are* cases in which habitual affective intentionality becomes solidified and thus epistemically problematic. Following Alia Al-Saji (2014) and her work on Merleau-Ponty and Fanon, I will call solidified emotion repertoires and an immovable way of habitual affective intentionality “affective ankylosis” and introduce it in the following.

In 2004 laicist France established the legal prohibition of wearing any religious symbols in school. Accordingly, Muslim girls were also not allowed to wear a headscarf anymore. Al-Saji (2014) describes the case of a girl searching for a compromise:

As the girl entered her classroom (in high-neck and bandana), the teacher responsible for the class reacted with immediate and visceral repulsion; she could not (physically and emotionally) tolerate the presence of the girl in her classroom, expressing violently and vocally her desire for the girl to leave. What was clear was that the teacher *saw* the girl *as* willfully incarnating religious dogmatism and gender oppression; her reaction was not that of *worked-through* argument or judgment, but of *prereflective* perception and *affect*. (2014: 134; emphasis added)

The task of the teacher would be to consider this situation carefully and to decide whether the frame of law is exceeded or not. But the teacher fails to enter a necessary understanding process in this situation because she discloses the girl as a specifically meaningful Gestalt:as embodying religious dogmatism and sexual oppression, as provoking, and as opposing rules and law at once. At this point, the crucial thesis is that the teacher is not able to disclose the girl *as another* possible meaningful Gestalt. She only has this very Gestalt within her emotion repertoire and this is the epistemic problem that becomes visible by emphasizing the socio-culturally specific habitualization.

The teacher does not reflectively judge that the girl is intolerable for any concrete reason. Rather, the intentionality and phenomenality of the affective reaction form an inseparable synthesis and cause the teacher to disclose the girl *instantaneously* as this specific Gestalt. Importantly, it seems like the appearance of the girl causes the disapproval of the teacher, but as Al-Saji argues, it is rather the other way around: the disapproval casts the clothing of the girl as not to be tolerated in the first place (Al-Saji 2014: 140). The girl is visually perceived and affectively disclosed at once as not to be tolerated. From the perspective of the teacher, this disclosure justifies – even more: naturalizes – the one-sided reaction of the disapproval itself (ibid.). From this perspective, it is not primarily the objects of affective intentionality which prescribe the disclosure, but the emotion repertoire of the feeling person. But *in their perception*, and this is epistemically problematic, the feeling person just reacts to “what is the case”. For the teacher, the girl *is* an expression of an oppression that is not to be tolerated. The especially problematic aspect thus is the *naturalization* and *rationalization* of affective disclosure of this one Gestalt. A person who offers a multitude of different ways of being and thus ways of being disclosed cannot but be perceived as this one Gestalt. This reduction is already epistemically problematic as such. The epistemic object (here: the person) is reduced to one feature and is arbitrarily ascribed meaningfulness. Humans, though, (and any other phenomenon towards which a subject takes an epistemic stance) can be reduced to *all sorts* of features. The meaningfulness of an object only occurs because of the differentiation – as Gestalt theory has already shown. The most basic form of meaningfulness in this sense is the difference between figure and ground. The naturalization of “*fixed* Gestalts” (like “dogmatic Muslima”) is made possible by the intentional structure of seeing and feeling and the dependence on such from habitualization processes, but it does not necessarily follow from it. One might think that in the repertoire of the teacher there are, like in the duck-rabbit picture, two possible Gestalts: the secular, Muslim woman who is expressing her emancipation by not wearing a headscarf; *or* the oppressed but herself fundamentalist woman who expresses her religious dogmatismby wearing a makeshift headscarf. The possibility of complex, manifold, and maybe contradictory forms of living of Muslim women does not fit this binary schema and remains a blind spot because of the teacher’s specific feeling alignment.

Rather than engaging in the affective work of responding to those lives, repulsion blocks that response, effectively congealing the porosity and fluidity of the affective sphere. Ethical unresponsiveness to the other (the perceptual and affective “I cannot”) is hence masked and justified by an affective hyperreaction that, at once sustains racialization and blocks the difficult work of responsivity by taking its place. This phenomenon can be described, following Fanon, as “affective ankylosis.” (Al-Saji 2014: 141)

It is plausible to assume that the girl, too, only has specific affective possibilities in her repertoire. It is far more likely that she will affectively react with shame, anger, or despair than with openness or benevolence. The teacher, by disclosing the girl “as x”, thus brings into existence this Gestalt not only for herself but for others as well. The affectedness of the teacher rebounds on the space of affectability of the girl – it gets inscribed into her lived body and leaves traces in her affective biography. The teacher’s racializing disclosure thus brings into existence a reality the girl cannot escape from and that sediments in her emotion repertoire, pre-structuring the way in which she, in turn, discloses meaningful Gestalts. For subjects who inhabit the world in this way, who are repeatedly exposed to such types of affections, the world is (not only) affectively one with a very specific space of possibilities. This holds in the same way for gender, disability, or whatever else is not the norm and thus stands out.

Importantly this does not only hold for racializing or objectifying disclosure. The ankylosis can be explained life-form-specifically and is transferable to any possible object that makes up such a form of living. These are sedimented such that humans directly and immediately disclose the Gestalt the meaning of which they have incorporated without conscious, reflexive evaluation. This is in general (epistemically) advantageous because it allows interacting appropriately in practice-specific contexts. Without this skilled dimension of affective interaction, humans would barely succeed in navigating sensibly with others through everyday life. But once affectivity gets *rigid*, the skillfulness of habitual affective intentionality is not innocent navigation anymore but becomes an unquestioned and unquestionable yet unreflected faith in things being right *this way*.^6^

## Epistemic Potentials of Affective Disruption

So far I introduced habitual affective intentionality and the potential epistemic *problems* arising from this way of disclosing meaningful Gestalts. In this section, I discuss ways to overcome these problems. I argued that habitual affective intentionality operates tacitly, i.e., the subject interacts in an undisturbed flow. Thus, *one* epistemic goal that my account brings to light is to make visible the contingency of a person’s *specific* affectability and their *specific* emotion repertoire. The affectively experienced disturbance of alignments can be *epistemically obstructive* if it leads to an attachment to these alignments and to a denial or other epistemically defective practices which allow one to maintain the stability of one’s little world(s). But an affectively experienced disturbance of order can also be *epistemically advantageous*. The moment of openness which comes with the disturbance does not *necessarily* lead to an attachment to alignment but rather *can* enable the subject to engage in an understanding process.^7^ This is connected to the *second* epistemic goal I elaborate on in this section, which is to acquire different access to the meaningfulness of complex phenomena – i.e., the ability to disclose a phenomenon as a different meaningful Gestalt. While the first epistemic goal is about getting aware of the own epistemic practices, the second one aims at a change in these and concerns a shift in perspective that can be achieved by leaving the own and travelling to other possible “worlds”.

### Affective Disturbance and Contingency

In the “good-bad-world” example above, it could also be the case that the child starts searching for explanations rather than supressing the negative feeling at the moment of disturbance. Their parent could impart accordingly that people are not necessarily *either* good *or* bad, but are rather many-faceted and contradictory. The child gets an explanation that does not shatter their old world but broadens it and differentiates the category of “bad humans” (Heller 1981: 62). With this example Heller demonstrates one of the epistemic functions of emotions she takes to be crucial, namely the alignment and realignment of knowledge (ibid.). The “fascist who is kind to children” acquires a non-distinct character for the affectively involved reading child and resists a distinct emotional evaluation of either aversion or devotion. While Heller sees the epistemic value of emotions in a new arrangement of epistemic objects, it seems to be more precise to say that a disturbance of alignment is caused by an unassimilable object and that this disturbance is experienced affectively. Such a disturbance enables a moment of openness that is necessary for being able to engage in understanding processes.

To illustrate this, recall the example from section two of Alex who visits Berlin and discloses the grey stone blocks they encounter against the practice of posting. Now consider the following continuation:

*After yesterday’s party, Alex is eating breakfast and scrolling on their tablet through diverse social networks. The usual pictures they acknowledge en passant. All of a sudden a bit of croissant sticks in their throat. Alex stares at the picture with the yoga pose they took yesterday.*

Together with many other selfies, the picture is published on a website called YOLOCAUST. At first glance, it shows colourful photos of happy people smiling into the camera. The photos contain likes, descriptions, and hashtags like #alemaniabonita or “#german gangster”. When the cursor scrolls over a selfie though, the colored picture gets black and white, and the background changes: There are no more grey stone blocks on which people strike fun, rather, they do so on dead bodies. The grey stone blocks – a holocaust memorial – are meant to commemorate exactly these bodies, victims of the national socialst mass murder. Two men who jumped from one stone to another now jump from one body to another in the photo-editing – the commentary on the photograph gets real – “Jumping on dead Jews”. This change, this transposition of reckless people practicing impression management on supposedly neutral stone blocks to pictures of abysmal inhumanity is *disruptive*.

*Alex’s gaze rests on the display. They are stock-still. Tears start to flood their eyes, they swallow. Everything within them convulses, they feel hot and cold. Their thoughts swirl while at the same time their head is empty. Alex gazes at their black and white selfie – sees themselves doing yoga on dead bodies: “Yoga is connection with everything around us”, they called the picture. Below it: 72 heart-emoticons from their followers. Alex remains paralyzed with shock.*

The memorial was made to remind people of the cruelty of the Holocaust and to make this accessible by its very specific architecture. It serves the purpose of commemoration and pausing. It should offer an epistemic contribution, to allow humans to come to grips with a very specific way of life and its abysses. It is oppressive, cold, cramped, inconvenient. But, for many people, it does not seem to transport the unspeakable horror of the holocaust. It does not affect them in *this way*. That many people picnic, skate, or do yoga on the memorial and first and foremost use it to produce post-able selfies shows that the meaningful Gestalt of the cruelty of the national-socialist reality is not disclosed. Rather completely different meaningful Gestalts are brought into existence – also for others. In his indignation and lack of understanding about this comportment at the memorial, the Israeli satirist Shahak Shapira initiated the yolocaust-project.^8^ His specific affective involvement – being intelligible against the background of his (affective) biography – is directed at the comportment of the people and their incomprehension of what this space symbolizes. With his photomontages, he allows the spectator access to what the memorial itself seems not to be able to deliver anymore. The disruption had an impact: Shortly after the publication of the YOLOCAUST-project all the pictures were removed from the website. The people depicted there could let their selfie be deleted once they recognized themselves there and were sure to be ashamed for taking the picture. The insight a spectator of the website could gain cannot be expressed in logical abstract constructs or simplified bullet points. It might be a mixed feeling of shame, shock, and grief which initiates a process of contemplation about one’s own comportment, societal structures, forms of living, the “Zeitgeist”, and the cruelty of the Holocaust, among other feelings.

This is an example of the epistemically positive effect of affectively experienced disturbance of alignment. For the person looking at the website, the transposition of the picture – and thus the change of a seemingly harmless context into absolute cruelty – elicits a disturbance of alignment and thus a way to be affected which again induces an understanding process towards a complex phenomenon. The affectively experienced disturbance thus *firstly* enables one to enter an understanding process regarding complex epistemic phenomena. *Secondly*, and this is at issue in this section, such an affectively experienced disturbance makes it possible to understand the contingency of one’s own *prereflexive affective habituality* as well as of one’s *emotion repertoire* – an understanding of the contingency of the affective alignment against the background of which meaningful Gestalts are disclosed. The affective disturbance that Alex experiences when they see themselves in the selfie allows them to access their very specific affectability, their very specific way of being affected by objects in their surroundings, and to radically disregard other possible disclosures of these objects. Against the background of the tacitly operating and taken-for-granted background of a central form of living making up their identity, the holocaust memorial appeared solely in the Gestalt of its “likes-creating-ability”. Because of their emotion repertoire and their affective habituality it was not possible for Alex to be affected by the stone blocks differently – as, for instance, someone who visits it in order to commemorate would be. In front of the very same object, this person discloses a completely different meaningful Gestalt and thus brings into existence a completely different reality for themselves and others. Emotions are not privately encapsulated phenomena – neither in their coming into existence nor in their effects. Shapira is outraged, I assume, exactly because the effects of the affectability of reckless selfie-crowds go beyond their individual leisure pursuit.

The insight Alex can gain is that their “little world” is fragile and that it could be very different. They *can* get access to the epistemic object “contingency of the specificity of the affectability”. My hypothesis is that the disturbance of alignment which is experienced affectively is a necessary but not sufficient condition for entering an understanding process. This claim does not presume anything about the duration of such a process and also nothing about the directions which this might take. All I have claimed so far is that it is a condition for the possibility of openness. The claim is not that someone who is deeply seated in a specific form of living can easily and encompassingly question it “from the outside” in this way. It remains true that it is the habitual patterns through which Alex disclosed their reality. And they are affectively attached to this alignment which they presumably desperately want to sustain. Giving it up would mean to lose the ability to adequately perceive specific practice-relevant cues and thus the ability to stay “in the game.”^9^ The moment of openness could only be very short – like in the case of the child in the scenario of denial or in the case of the fashion blogger who searches for an alternative explanation after a justification for the old alignment and switches channels. The child can immediately decide to call the book stupid – the understanding process is directly cut off again. Or the child could initially engage in an understanding process but then forget the insight about the inadequacy of their black-white-worldview, or the insight could not make it into the repertoire of the child, because it does not get practiced, enforced, or rewarded socially. How long subjects stay in the attitude of openness and are able to dwell on the question, in a state of “non-alignment” and indetermination can vary greatly. The affectively experienced disturbance initially dis-orders – it operates privatively (it bereaves order) not positively (endowing order); that means it is effective by taking away the alignment and thereby allows for an openness for other affectabilities and Gestalts to be disclosed.

### Disturbance and Transformation of Alignments: “World-Travelling”

If habitual affective intentionality, the emotion repertoire, and the habitual lived body already determine the space of what is felt and thus understandable – how is it then possible to *change* these ways of disclosure? What my account brings to light is that specific epistemic positions are *not* changeable by argument or discourse: “[P]eople don’t change their beliefs, especially ones that serve their interests, by being lectured at” (Haslanger 2015). I will make plausible the claim that an epistemic transformation of habitual affective intentionality, of the emotion repertoire, and the habitual lived body can be enabled by affective *disruptions* which temporarily and partially allow us to “travel other worlds” (Lugones 1987). That means comprehension beyond mere recognition of already familiar Gestalts becomes possible. What is disrupted and potentially transformed are the habitual schemata themselves *through which* the world is disclosed.

Suppose Anniken, the fashion blogger, would not switch channels and deny problems in the textile industry but would search for explanations, would do some research, and thus enter an understanding process. The epistemic value of the affectively experienced disturbance would thus already initiate *epistemic exploration* and *increased attention* to the epistemic object “fashion industry” and temporarily would make *suppression and denial impossible*. A person may understand *that* “it is bad” for refugees to leave their families and homes and to lose their whole lives, but if that does not affect them, if they can just put this information aside, they do not disclose the phenomenon as a meaningful Gestalt. Goldie calls this phenomenon “knowing-but-not-knowing” (2008: 159) and denotes an intrinsic intertwining of epistemic failure and akrasia: “One might put it like this: if we *really* knew how terrible the genocide was, then we would try to do something about it” (ibid.). Anniken *knows* in the same sense *that* the fashion industry is built upon exploitation and that she engages in this actively. But she does not understand the meaningfulness of this circumstance if she does not disclose it affectively. This cannot be changed by argument. One possible way is a cracking of the ankylosis and attachment to alignment and form of living which is enabled that way. Consider the following scenario:

*Anniken travels to Cambodia in order to get insight into the production conditions of the clothes she buys and advertises. She lives together with a sewer and works with her in a factory – everything is filmed for a documentary.* 10 *At the beginning Anniken says “They have a straight job. They have a job – at least!” Then she works and cannot believe how hard the work actually is; how grueling, humiliating, monotonous – plainly horrible this is. “I thought you were kidding when you said we would sit there for hours.” Her power vanishes. Also, leisure time is not better. She shares a small room with four others, they sleep like sardines packed in a can. There is not enough money for food and the transportation to work is like animal transportation in a truck with mask as protection against the polluted air. Anniken cannot stand it anymore and ultimately collapses. Her “They have a straight job. They have a job – at least!” transforms into a “speechless” “What kind of life is that?”.*

Anniken finds herself with her habitual lived body and emotion repertoire in a radically different little world, a little world she cannot engage with skillfully according to the habitual normative structure to disclose meaningful Gestalts. All alignments allowing her to skillfully engage in her familiar world break down and she loses orientation. This loss of alignment is unbearable and leads to break-down.

The disruption allows access to the world of the sewer she lives with. What Anniken experiences *can* make her understand what it means to be a different part of the complex phenomenon of the textile industry – not the selling and advertising the products, being liked for wearing and making money with them, but their production. Anniken does not “know” this in the sense of a supposedly unaffective knowledge and also not only acquires one new quale of, say, sitting at a sewing machine for hours. Rather she understands *what it means to live that very different form of living*. If Anniken changes her epistemic position from “They have a straight job. They have a job – at least! “ to “What kind of life is that? “ she does not only change single aspects in her complex “frame of mind”. Rather the whole Gestalt changes, which again leads to changes in comportment, narratives, and practices. It is thus possible to get access to other forms of living, to other ways of being in the world, by a temporary loosening of affective ankylosis and affective attachments to life form specific alignments. Although this surely includes that Anniken may also close this possibility again.^11^

Three remarks are necessary at this point: (1) It is likely that Anniken will return to her old habits of disclosure. This shows that it is necessary to think of disruptability as an ethico-epistemic *ability* that needs cultivation in order to be sustained. (2) Furthermore, for "world travel" in Lugones’ sense, it is not necessary to really travel to other countries, as in the example. Rather, subjects can also travel to the world of their mother, their neighbor, or another form of living by letting themselves be moved by what moves the other, by taking their perspective, by allowing the world to affect them in the way it affects other ways of being. And (3) this example contains the problematic aspect of what one might call “poverty porn” and the tendency of privileged members of western societies to visit poor countries in a voyeuristic manner for the sake of adventure and not at all considering the structural violence and harm they are allegedly shocked about. These three remarks help to be explicit about what is and what is not claimed by proposing affective disruptability and “world-travelling” to be a potential ehtico-epistemic ability. It is a *potential* epistemic gain to confront oneself with the difference of other worlds, but the very context of this travel plays a crucial role as well as the willingness to cultivate the transformed repertoire and the plastic affectability. That there is a significant danger of repression and even of fostering old and habitual patterns of disclosure needs to be taken seriously and is in need of further research.

## Conclusion and Outlook

In order to engage in understanding processes, I argued in this paper, we need to consider the affective relevance for the specificity of disclosed meaningful Gestalts, for the “little worlds” which are inhabited and the practices making up humans’ identities. It is not just single beliefs that can be taught and changed by argument, but rather many beliefs belonging to a network of concerns relevant for forms of living to be upheld. If we do not understand the intertwining of affectivity and these meanings, we cannot establish the often urgently needed “sensemaking overlap” Varela had in mind when considering the Chilean Civil war being caused by such an epistemic problem. I do not claim that affective disturbances and disruptions are sufficient to transform emotion repertoires and make visible the contingency of habitual affective intentionality. By pointing out these ways of changing epistemic positions, I rather want to put emphasis on the importance of flexible, agile affectability and a plastic emotion repertoire and to point at the solidified ways of making sense of the world and the incapability of subjects to enter understanding processes calling into question the familiar, the comfortable, the habitual. Being stable and centered as a person though is a necessary condition in order to cultivate the capacity to let oneself be disrupted without the fear of losing all ground. Thus, what I have established here belongs to a more encompassing need in terms of education – educating ourselves to be valuable beings independent of strict identities and strict forms of living. If these are harmful to ourselves or others we should be able to give them up without getting into existential troubles. I argued that what I call affective disruptability and accordingly “world-travelling” can be seen as *(ethico-)epistemic abilities* which can be cultivated. I thus plea for cultivation of a receptibility for difference, for cultivation of an adaptable and malleable lived body, of a pluralistic emotion repertoire, and of the capability to disclose meaningful gestalts affectively, to integrate them into understanding processes, and to call them into question. This requires work though, firstly of the individual who needs a willingness for openness. Secondly, societal practices in the form of art for instance are needed to foster and materialize a possibility to embrace difference. What exactly this epistemic ability – as potential—is about and what is needed to cultivate it requires further elaboration and research.

## Footnotes

1 “Varela shows that Chile had become polarized into two separate worlds without communication, two autonomous systems with no sensemaking overlap, no means of mutual recognition.” (Protevi 2009): 44).

2 Here and in the following, I am drawing on material from von Maur (2018, 2021).

3 I elaborate on material from von Maur (2018) in the following.

4 Following Alia Al-Saji, who uses the concept in the context of racializing perceptual schemas, I “use the term *unassimilable* here following Merleau-Ponty […T]he unassimilable is that which cannot fit within racializing schemas and hence poses a difficulty for the racializing imaginary” (Al-Saji 2014: 171, Fn. 91). I do not restrict my analysis to racializing alignments of affectivity, however.

5 Judith Butler (1997: 6ff.) uses the term “passionate attachments” to consider the process of becoming a subject within dependence relations.

6 Bourdieu calls the skillful dimension the *practical sense* and the unquestioned faith *doxa*. For an elaboration on this see von Maur (2018).

7 The “can” is decisive: It does not have to, but can; thus, it is a necessary but not sufficient condition. I do not claim to offer a “great solution” or method but suggest that without disruption of alignments there is no openness. This is meant analytically and not so much as an empirical claim. Also, subjects with a high change-ability have to (analytically speaking) become disturbed in their alignment because sustaining the alignment is standstill and thus leaves the alignment unmoved. A strong ankylosis makes a willingness for change much harder to achieve. We believe in things *because* they sustain our narratives, realities, and practices.

8 The title is a portmanteau word, consisting of the acronym YOLO and the word “Holocaust”. YOLO stands for “You Only Live Once” and denotes a lifestyle according to which the individual should enjoy any present moment of life, should seek adventures and risks. This is an attitude of youth and exhibits a certain infantilization and gamification of life I would rather deem an instance of escapism than of real joy and fulfillment in the original “carpe diem” way of living.

9 For an elaborate account of pro and contra arguments of “evading discomfort” in the context of moral and political implications of implicit bias, see the work of Ditte Marie Munch-Jurisic (2020a, 2020b).

10 This example considers the web documentary “Sweat Shop – Deadly Fashion” (2014) from Joakim Kleven, commissioned by the Norway newspaper “Aftenposten”. Anniken Jørgensen and two other fashion bloggers from Norway (about 20 years old) are sent to Cambodia for this documentary to visit the textile factory producing their favorite clothes. The documentary has been watched more than 1.5 million times and caused a discussion about exploitation and working conditions in the textile industry.

11 Regarding the question of which epistemic value emotions exhibit when it comes to the entrance of other “moral terrains” (Figueroa and Waitt 2008), see Dotson and Whyte 2013.
